# Carbon monoxide therapy protects against hepatic microvascular injury in a mouse model of murine hemorrhagic shock and resuscitation

**DOI:** 10.1186/cc10871

**Published:** 2012-03-20

**Authors:** H Gomez, I Nassour, P Loughran, J Brumfield, L Otterbein, B Zuckerbraun

**Affiliations:** 1 University of Pittsburgh, PA, USA

## Introduction

The purpose of this study is to evaluate the effects of inhaled carbon monoxide (CO) as an adjunct to resuscitation on hepatic microvascular and endothelial integrity in a murine model of hemorrhagic shock and resuscitation (HSR). Others and ourselves have previously demonstrated that CO can protect against organ injury in experimental models of HSR [1]. Additionally, CO can prevent tissue hypoxia during hemorrhage. Based upon this we hypothesized that CO prevents hepatic injury and prevents hepatic hypoxia by maintaining endothelial integrity and the hepatic microvascular circulation.

## Methods

Male C57BL/6 mice underwent sham operation or hemorrhage to a target MAP of 25 mmHg. Mice were maintained at this pressure for 120 minutes and then resuscitated with Ringer's lactate at two times the volume of total shed blood. Mice were sacrificed 4 hours after resuscitation. Mice were randomized to receive room air or inhaled CO (250 ppm) for 30 minutes starting 90 minutes into the shock period (*n *= 6 to 8 per group). Relative hepatic hypoxia was determined using EF5 immunofluorescence. Sinusoidal integrity was determined by scanning electron microscopy of the hepatic sinusoidal endothelium and Evan's blue tissue levels. Leukocyte stasis, rolling, and adhesion were determined using intravital microscopy of post-sinusoidal hepatic venules. Statistical analysis was determined by ANOVA.

## Results

EF5 staining demonstrated that hemorrhagic shock induced liver hypoxia, which was prevented by CO treatment. Scanning EM imaging of hepatic sinusoids demonstrated that HSR results in loss of normal endothelium, with loss of fenestrations, rounding of cells, and adherent circulating cells. CO therapy prevented these changes. Relative hepatic levels of Evans blue, suggesting endothelial leak, were increased 1.7 ± 0.23-fold in HSR compared to sham-operated mice (*P *< 0.05). CO treatment minimized endothelial leak, resulting in a 1.23 ± 0.21-fold increase compared to sham (*P *< 0.05 compared to air-treated HSR). In addition, leukocyte rolling and adhesion were significantly diminished by CO as compared to the air-treated group in HSR.

## Conclusion

CO protected the hepatic sinusoidal endothelium from HSR-induced injury. Further investigations into the mechanisms of action are necessary. CO therapy may prove to be a useful resuscitative adjunct in the treatment of HSR.
